# Voice disorder classification using convolutional neural network based on deep transfer learning

**DOI:** 10.1038/s41598-023-34461-9

**Published:** 2023-05-04

**Authors:** Xiangyu Peng, Huoyao Xu, Jie Liu, Junlang Wang, Chaoming He

**Affiliations:** School of Mechanical Engineering, Southwest Jiaotong University, Chengdu, 610031 China

**Keywords:** Data processing, Machine learning, Predictive medicine

## Abstract

Voice disorders are very common in the global population. Many researchers have conducted research on the identification and classification of voice disorders based on machine learning. As a data-driven algorithm, machine learning requires a large number of samples for training. However, due to the sensitivity and particularity of medical data, it is difficult to obtain sufficient samples for model learning. To address this challenge, this paper proposes a pretrained OpenL3-SVM transfer learning framework for the automatic recognition of multi-class voice disorders. The framework combines a pre-trained convolutional neural network, OpenL3, and a support vector machine (SVM) classifier. The Mel spectrum of the given voice signal is first extracted and then input into the OpenL3 network to obtain high-level feature embedding. Considering the effects of redundant and negative high-dimensional features, model overfitting easily occurs. Therefore, linear local tangent space alignment (LLTSA) is used for feature dimension reduction. Finally, the obtained dimensionality reduction features are used to train the SVM for voice disorder classification. Fivefold cross-validation is used to verify the classification performance of the OpenL3-SVM. The experimental results show that OpenL3-SVM can effectively classify voice disorders automatically, and its performance exceeds that of the existing methods. With continuous improvements in research, it is expected to be considered as auxiliary diagnostic tool for physicians in the future.

## Introduction

Vocal organs serve as crucial communication tools for human beings, enabling us to convey information and express emotions. In today's society, approximately one-third of workers, such as teachers, broadcasters, singers, and telephone operators, rely on their voices as their primary tools^1^. Unfortunately, many people suffer from voice disorders due to improper vocalization, overuse of their voices, or colds. Symptoms can include voice fatigue, difficulty speaking, or abnormal pitches^2^. When the voice is compromised, it can greatly impact an individual's daily life.

Voice disorders are very common diseases, especially for people who are vocal for a long time at work. A survey conducted across 27 states in Brazil revealed that 33.9% of teachers reported experiencing voice disorders, and 55% had to take time off work due to voice-related problems^3^. Another study involving 573 teachers in Salvador, Brazil, found that 23% experienced temporary aphonia, and 12% had vocal cord nodules^4^. Nelson et al.^5^ surveyed 2401 randomly selected participants, including 1243 teachers and 1279 nonteachers, and found that 57.7% of teachers had a voice disorder, while only 28.8% of nonteachers had ever been ill with this disease. In traditional clinical diagnosis cases, a variety of medical examinations, such as laryngoscopy, stroboscopy, and endoscopy, are required to diagnose voice disorders. These tests must be performed by professional doctors with specialized equipment, and these tests are often invasive, time-consuming, expensive, and painful for the patients. The shortcomings of traditional diagnosis methods make many patients reluctant to go to professional institutions for examinations or treatments, resulting in delays in receiving optimal treatments. A survey found that 29.9% of the participants had suffered from a voice disorder, but only 5.9% had sought professional treatment^6^.

To facilitate the convenient and rapid diagnosis of voice disorders, acoustic analysis systems have been developed and utilized in clinical settings, such as the multidimensional voice program (MDVP)^7^, Praat^8^ and Vox4Health^9^. These systems can be used to extract voice feature parameters, including the harmonics-to-noise ratio, fundamental frequency, normalized noise energy, jitter and shimmer. However, these systems cannot automatically evaluate the extracted parameters; an experienced doctor is still required to evaluate these parameters to work out a diagnosis.

In recent years, with the rapid development of computer technology and the accumulation of big data, machine learning has demonstrated its excellent performance in many fields^10–12^, and many researchers have also tried to apply it to the recognition of voice disorders and have achieved good results. For example, Leung et al.^13^ classified samples into healthy or pathological states using an SVM, achieving an accuracy of 69.3%. Laura et al.^14^ extracted F0, jitter, shimmer and HNR as features and then used an SVM, a logistic model tree, a Bayesian classifier and a decision tree for classification purposes, finally obtaining approximately 86% accuracy. Chen et al.^15^ used the Hilbert-Huang transform (HHT) to extract features from sound signals and classified them based on k-nearest neighbors (KNN), achieving an accuracy of 93.3%. Cordeiro et al.^16^ performed pathological identification based on the first peak of the spectral envelope for pathological voice characterization, achieving an accuracy of 94.2%. Chen et al.^17^ used the MFCC and a DNN for voice disorder classification. Mittal et al.^18^ classified voice disorders by integrating multiple classifiers. Mittal et al. proposed a noninvasive voice pathology recognition framework by fusing deep learning with nonparametric learners at the decision level. Kwok et al.^19^ proposed a combination of generative adversarial networks and fuzzy C-means clustering (CGAN-IFCM) for the multiclass recognition of voice disorders. Although the above research works have achieved good results, some shortcomings remain. (1) Traditional machine learning algorithms heavily rely on artificial feature engineering to extract meaningful features, which usually requires considerable expertise and experience. (2) Feature selection often relies on expert experience and subjectivity, which can lead to information loss and bias. (3) The direct application of deep neural networks is very difficult due to the small amount of available medical data.

Different from the traditional machine learning and deep learning, model-based transfer learning (model-TL) can transfer knowledge learned from the source domain to the target domain without requiring a large number of samples for training. Many researchers have applied model-TL in the field of pathology diagnosis and have achieved good results. For instance, Karaman et al.^20^ developed a deep convolutional neural network (CNN) classifier based on model-TL that can identify Parkinson's patients by utilizing sustained vowels as voice biomarkers. Weimann et al.^21^ introduced model-TL to classify heart rhythms from short ECG recordings. By pretraining the model on a large-scale dataset, the performance of the CNN on the target dataset was improved by 6.5%. Shi et al.^22^ combined the VGGish network with a bidirectional gated recurrent unit neural network, used a large-scale audio set to train the VGGish network, transferred the network parameters to the target network for lung disease recognition, and improved the recognition accuracy achieved for lung sounds. Georgopoulos et al.^23^ presented an algorithm for pathological voice detection based on advanced time–frequency signal analysis and transfer deep learning.

Inspired by transfer learning, we propose a novel model-based transfer learning framework for multiclass voice disorder classification. The OpenL3-SVM framework is constructed with the pretrained OpenL3 CNN and a top SVM classifier. The OpenL3 network is a feature extractor that can extract high-level feature representations from original voice signals. A fine-tuned training strategy is proposed to make the pretrained model more adaptable to the target task while retaining the source domain knowledge. The SVM classifier is connected to the top of the OpenL3 network for voice disorder classification, thereby improving the performance of the network on small-scale target datasets. The contributions of this paper are as follows.

1. A novel model-based deep transfer learning framework is proposed for multiclass voice disorder classification.
2. To overcome the issue of data shifting between the source and target domains, different transfer learning strategies are proposed to improve the performance achieved by the model on the target task.
3. The experimental results show that the proposed method can effectively identify specific types of disorders. Additionally, compared with the existing advanced methods, the proposed method achieves better performance.

**Figure 1:**
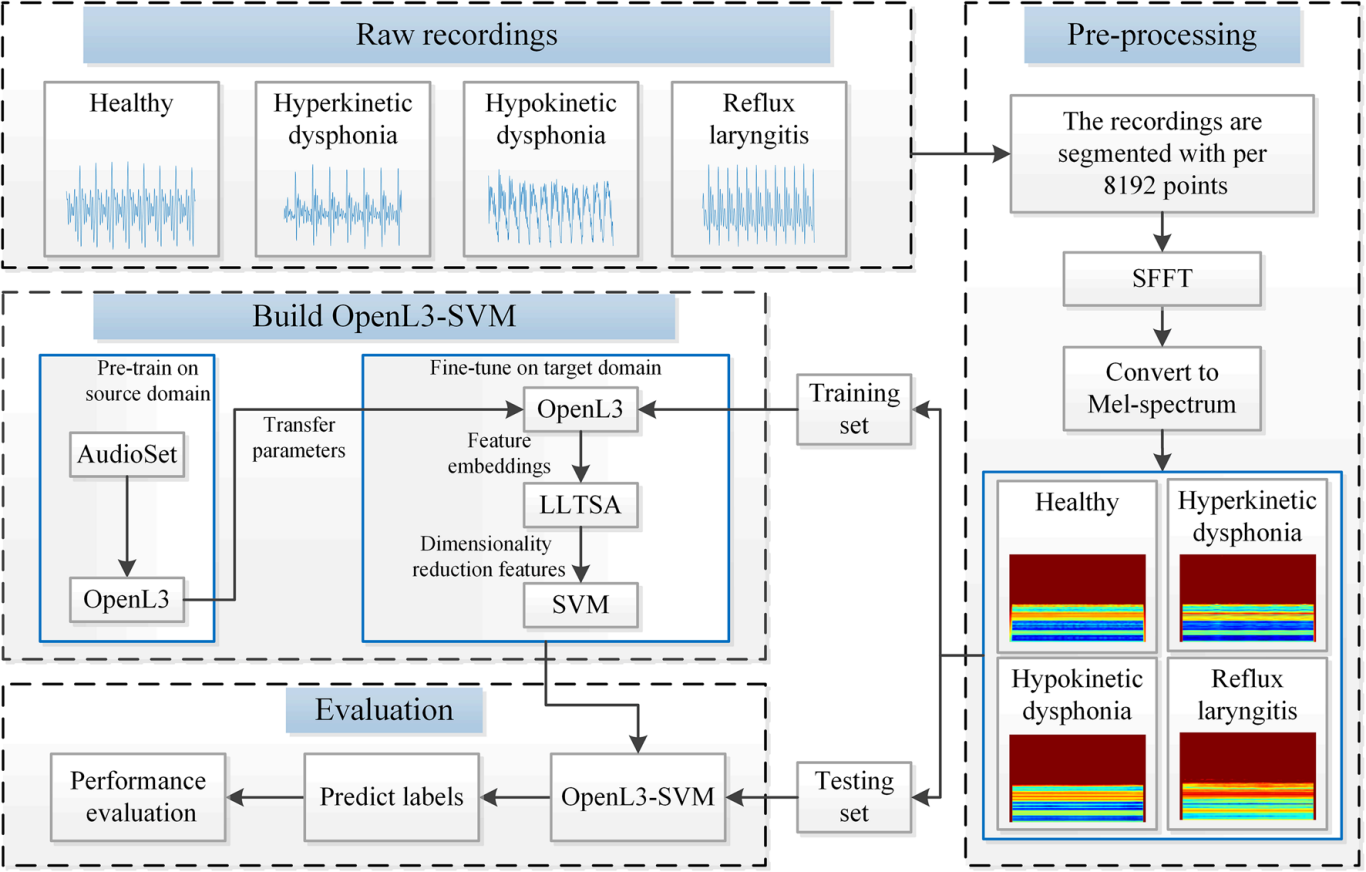
Flowchart of the proposed method.

The structure of this paper is as follows. The proposed OpenL3-SVM network framework is shown in "Methods" section. The experimental results and discussion are presented in "Results" section. The discussion part of the research work is presented in "Discussion" section. The conclusions of the research work are presented in "Conclusion" section. The specific process of the proposed method is shown in Fig. 1.

## Methods

### Data descriptions and experimental environment

The voice recordings used in this paper were obtained from the Voice ICarfEDerico II Database (VOICED)^24,25^. The VOICED contains 150 pathological samples and 58 healthy samples. The subjects ranged in age from 18 to 70 years and included 135 females and 73 males. The 150 pathological recordings included 72 hyperkinetic dysphonia recordings, 40 hypokinetic dysphonia recordings and 38 flux laryngitis recordings. The diagnosis process is based on the SIFEL clinical standards proposed by the Italian Society of Phoniatrics and Logopaedics^26^. All records were collected through the m-health system installed on a Samsung Galaxy S4. The device was positioned at 45° and 20 cm away from each subject during the recording procedure. The sampling frequency of the system was 8000 Hz, and the resolution was 32 bits. During the acquisition stage, each subject was instructed to continuously pronounce the vowel 'a' at a constant sound intensity, with each recording lasting 5 s. The published recordings were filtered with appropriate filters to remove the noise contained within them.

This experiment is implemented based on MATLAB R2022a. The hardware resources include an AMD 3700 × CPU, a 12-GB NVIDIA RTX 3060 GPU, and 32 GB of RAM.

### Mel spectrogram

This paper uses a Mel spectrogram as the input of OpenL3. A Mel spectrogram is a logarithmic frequency spectrum under the Mel scale. The Mel scale imitates the human ear's perception of sound and weakens the perception of high-frequency signals. To obtain the Mel spectrogram, it is first necessary to resample the original signals to 48 kHz, perform framing and use a Hamming window for windowing. The frame length is 2048 points with an overlap length of 1806. The time domain signals are converted to the frequency domain using the short-time Fourier transform (STFT)^27^.

Then, the frequency bands of each frame are calculated using a Mel filter bank. The Mel filter bank is a filter bank composed of multiple triangular filters; it can smooth the spectrum and reduce the data quantity. Mel filter banks can be composed of equal-area filters or equal-height filters. The latter type pays more attention to low frequencies and is typically used for the processing of vocal signals. The transfer function of the filter is:

1 $${H}_{m}(k)=\left\{\begin{array}{ll}0, & \quad k<f(m-1)\\ \frac{k-f(m-1)}{f(m)-f(m-1)},& \quad f(m-1)\le k\le f(m)\\ \frac{k-f(m-1)}{f(m)-f(m-1)},& \quad f(m)\le k\le f(m+1)\\ 0, & \quad k>f(m+1)\end{array}\right.$$

In Eq. (1), *k* is the frequency point obtained after executing the STFT; $m$ is the serial number of the filter; and the center frequency *f*(*m*) corresponding to the filter is calculated with the following formula:

2 $$\begin{array}{c}f\left(m\right)=\left(\frac{N}{{f}_{s}}\right){F}_{mel}^{-1}\left({F}_{mel}\left({f}_{l}\right)+m\frac{{F}_{mel}\left({f}_{h}\right)-{F}_{mel}\left({f}_{l}\right)}{M+1}\right)\end{array}$$

3 $$\begin{array}{c}{F}_{mel}(f)=1125\mathrm{ln}\left(1+\frac{f}{700}\right)\end{array}$$

4 $$\begin{array}{c}{F}_{mel}^{-1}\left(b\right)=700\left({e}^{b/1125}-1\right)\end{array}$$

In Eqs. (2)–(4), ${f}_{l}$ and ${f}_{h}$ represent the lowest and highest frequencies of the filter frequency range, respectively; $M$ is the number of triangular filters; $N$ is the length of the Fourier transform; ${f}_{s}$ is the signal sampling frequency; $b$ is the true frequency.

Finally, the result of the Fourier transform is multiplied by the filter bank to obtain Mel bands, and the Mel bands of each frame of the signal are obtained. To simulate the nonlinear perception of sound by humans, we implement a logarithm with a base of 10 after square rooting the filtered result and subtract the maximum value to obtain the final input.

### Construction of the OpenL3-SVM network

**Figure 2:**
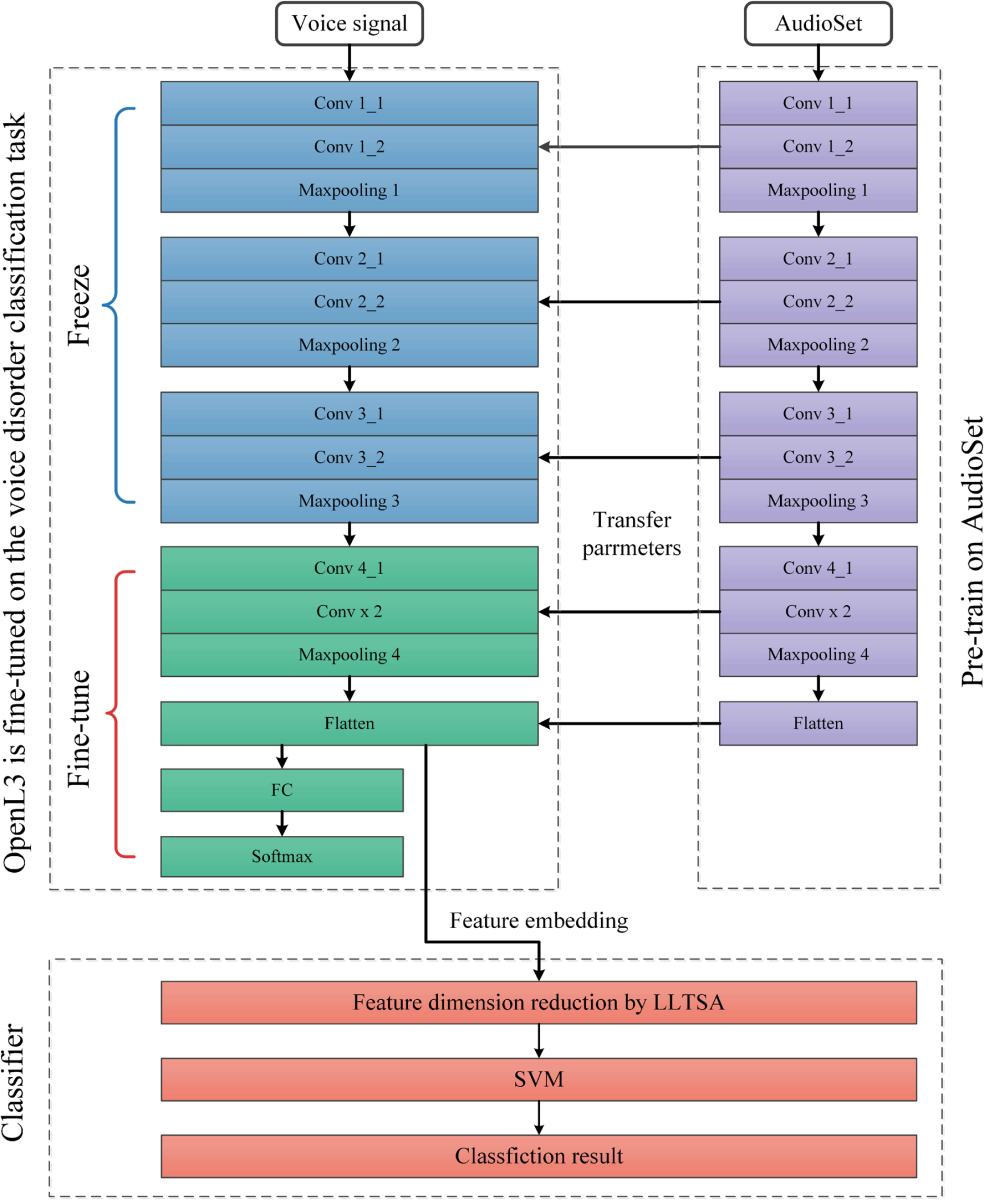
OpenL3-SVM network structure.

The OpenL3 network was proposed by Cramer et al. in reference^28^. The author improved audio tasks based on L3-Net^29^ and used Audio Set^30^ for pretraining with the unsupervised training mode (similar to L3-Net). Several studies have demonstrated the effectiveness of the OpenL3 network. Therefore, we propose a novel multiclass model for voice disorder recognition by combining OpenL3 with an SVM, and the structure and parameters of the network are shown in Fig. 2 and Table 1. By transferring the pretrained OpenL3 model, the generalization ability of the network for few-shot tasks is improved. Furthermore, as a classic classifier, the SVM has good performance and robustness in few-shot tasks.

**Table 1:** Parameters of the OpenL3 network.

Layer	Kernel size	Stride	Padding	Output size
Input	–	–	–	128 × 199 × 1
Conv 1_1	3 × 3	[1 1]	Same	128 × 199 × 64
Conv 1_2	3 × 3	[1 1]	Same	128 × 199 × 64
Max pooling 1	2 × 2	[2 2]	[0 0 0 0]	64 × 99 × 64
Conv 2_1	3 × 3	[1 1]	Same	64 × 99 × 128
Conv 2_2	3 × 3	[1 1]	Same	64 × 99 × 128
Max pooling 2	2 × 2	[2 2]	[0 0 0 0]	32 × 49 × 128
Conv 3_1	3 × 3	[1 1]	Same	32 × 49 × 256
Conv 3_2	3 × 3	[1 1]	Same	32 × 49 × 256
Max pooling 3	2 × 2	[2 2]	[0 0 0 0]	16 × 24 × 256
Conv 4_1	3 × 3	[1 1]	Same	16 × 24 × 512
Conv 4_2	3 × 3	[1 1]	Same	16 × 24 × 512
Max pooling 4	16 × 24	[16 24]	Same	1 × 1 × 512
Flatten	–	–	–	512

1. Input layer
  The OpenL3 network accepts three different input types: linear, Mel 128, and Mel 256 spectrograms. Here, 128 and 256 denote the number of Mel filters. In Cramer's study (cited as reference^28^), the impacts of these inputs on embedding performance was investigated. The experimental results revealed that Mel spectrograms outperformed linear spectra. Mel 256 performed slightly better than Mel 128, but its larger number of filters also required a larger amount of data, which greatly reduced the training speed. Consequently, we use Mel 128 as the input feature with input layer dimensions of 128 × 199. Batch normalization is applied after the input layer.
2. Convolutional layer
  The convolutional layer is the core component of a CNN^31^. This layer employs multiple convolution kernels to extract local features from the input and gradually scans with a window to extract the features of all inputs. The scanning range of the convolutional layer window is called the receptive field. The convolutional layer design of OpenL3 is similar to that of VGGNet, with a 3 × 3 receptive field for the convolution kernel and a sliding window stride of 1. The operation process of the convolutional layer can be described by Eq. (5):
  5 $$\begin{array}{c}c(x,y)=F\left(\sum_{n=0}^{N-1}\sum_{m=0}^{M-1}{w}_{n,m}\cdot \varphi \left(x+n,y+m\right)n+b \right)\end{array}$$
  where N and M represent the length and width of the convolution kernel, respectively; ${w}_{n,m}$ represents the corresponding weights of the convolution kernel at position (n, m); φ denotes the feature of the output of the previous layer, $b$ is the bias, and $F\left(\cdot \right)$ represents the activation function. The activation function used in this network is a rectified linear unit (ReLU). The ReLU can bring a certain sparsity to the network and prevent gradient dissipation. Its expression is given in Eq. (6):
  6 $$\begin{array}{c}F\left(x\right)=\mathrm{max}\left(0,x\right)\end{array}$$
3. Pooling layer
  CNNs generally consist of multiple convolutional layers, and pooling layers are often used after convolutional layers. These pooling layers can extract the essential information from the input features and reduce the required amount of data. Additionally, pooling layers help to mitigate overfitting, which is a common issue in deep CNNs. The pooling layers commonly used by CNNs include max pooling and mean pooling layers. OpenL3 utilizes max pooling layers, wherein the features are extracted by gradually scanning the window and selecting the maximum value within the window range. The pooling layer of the OpenL3 network employs a window size of 2 × 2, a stride of [2 2], and padding of [0 0 0 0].
4. Batch normalization
  During the training process, the data distribution may shift or transform as the network becomes deeper. This data distribution shift becomes more pronounced as the network architecture becomes more complex. As a result, during backpropagation, the gradient of the lower neural network dissipates, slowing down the network convergence rate. Batch normalization (BN) addresses this issue by adjusting the data distribution through specific normalization methods, thus maintaining the network convergence speed and mitigating overfitting. The calculation steps for batch normalization are as follows.
  Step 1: Calculate the mean value ${\mu }_{X}$ of each feature element in the given minibatch, as shown in Eq. (7):
    7 $$\begin{array}{c}{\mu }_{X}=\frac{1}{n}\sum_{i=1}^{n}{x}_{i}\end{array}$$
    where batch X = [${x}_{1}$, ${x}_{2}$,…, ${x}_{n}$ ], and ${x}_{i}$ represents a sample. $n$ represents the minibatch size.
  Step 2: Calculate the variance value ${\sigma }_{x}^{2}$ of the minibatch, as shown in Eq. (8):
    8 $$\begin{array}{c}{\sigma }_{x}^{2}=\frac{1}{n}\sum_{i=1}^{n}{\left({x}_{i}-{\mu }_{x}\right)}^{2}\end{array}$$
  Step 3: Calculate the normalized element ${\widehat{x}}_{l}$ using the variance and mean, as shown in Eq. (9):
    9 $$\begin{array}{c}{\widehat{x}}_{l}=\frac{{x}_{i}-{\mu }_{x}}{\sqrt{{\sigma }_{x}^{2}+\upvarepsilon }}\end{array}$$
    where ε is a constant used to prevent the denominator from being 0.
  Step 4: Apply a scale and offset operation to the regularized data obtained in step 3 to obtain the output ${y}_{i}$.
    10 $$\begin{array}{c}{y}_{i}=\gamma {\widehat{x}}_{i}+\beta ={BN}_{\gamma ,\beta }\left({x}_{i}\right)\end{array}$$
    In Eq. (10), γ is the scale factor, and β is the shift factor. These two parameters need to be learned during training.
    The OpenL3 network uses batch normalization after the input layer and before all activation layers.
5. Classification layer
  The original OpenL3 network uses Softmax as a classifier, and its original classification layer is removed to extract feature embeddings. Softmax is replaced with an SVM^32^ to accommodate few-shot learning tasks. SVMs is a very classic classification algorithm that always achieve good performance and robustness in many tasks, especially in few-shot tasks.
  OpenL3 extracts 512-dimensional features from the original signal, but many of these features have little effect on the classification results. To speed up the training process, the output of OpenL3 uses LLTSA for feature dimensionality reduction. Since the SVM is very sensitive to the magnitudes of features, the features obtained after dimensionality reduction are normalized.

### Performance evaluation indicators

To quantitatively evaluate the performance of the proposed method, four indicators, including F1, accuracy (ACC), sensitivity (SEN) and specificity (SPE), are adopted. The four indicators are denoted as follows:

11 $$F1 = \frac{{2 \times \overline{TP} }}{{m + TP - \overline{TN} }}$$

12 $$Accuracy = \frac{{\overline{TP} + \overline{TN} }}{{\overline{TP} + \overline{TN} + \overline{FP} + \overline{FN} }}$$

13 $$Sensitivity = \frac{{\overline{TP} }}{{\overline{TP} + \overline{TN} }}$$

14 $$Specificity = \frac{{\overline{TN} }}{{\overline{TN} + \overline{FP} }}$$

In Eqs. (11)–(14), TP and TN refer to the numbers of correctly identified positive and negative samples, respectively, while FP and FN represent the numbers of incorrectly identified positive and negative samples, respectively, and m represents the total number of samples. In multiclassification tasks, TP, TN, FP, and FN cannot be directly calculated, so micro-averaged evaluation criteria are adopted in this paper. First, each class is considered positive, and the others are considered negative to calculate the four confusion matrices. Then, the average of all confusion matrices is calculated to obtain $\overline{TP}$, $\overline{TN}$, $\overline{FP}$ and $\overline{FN}$. Finally, the average values are used to calculate each evaluation indicator. To verify the stability of the model, the standard deviation of each indicator is also calculated and recorded in the following format: indicator ± standard deviation.

## Results

### Data preprocessing

All 208 records in the database are used in this paper, and each recording has a single channel and contains approximately 38,000 points. Each record has approximately 0.15 s at the beginning where no sound is acquired. To prevent this anomalous segment of records from interfering with the network training process, we remove the 0.15 s at the beginning of each record. Then, each record is segmented with a length of 1.024 s, and each sample contains 8192 points. We use the sliding window technique to expand the samples, with an overlap of 4096 points between adjacent windows. In addition, to prevent the data imbalance from impacting the results, we use a random downsampling technique to create a balanced subset. The number of expanded samples is 1040, where each class contains 260 samples.

**Figure 3:**
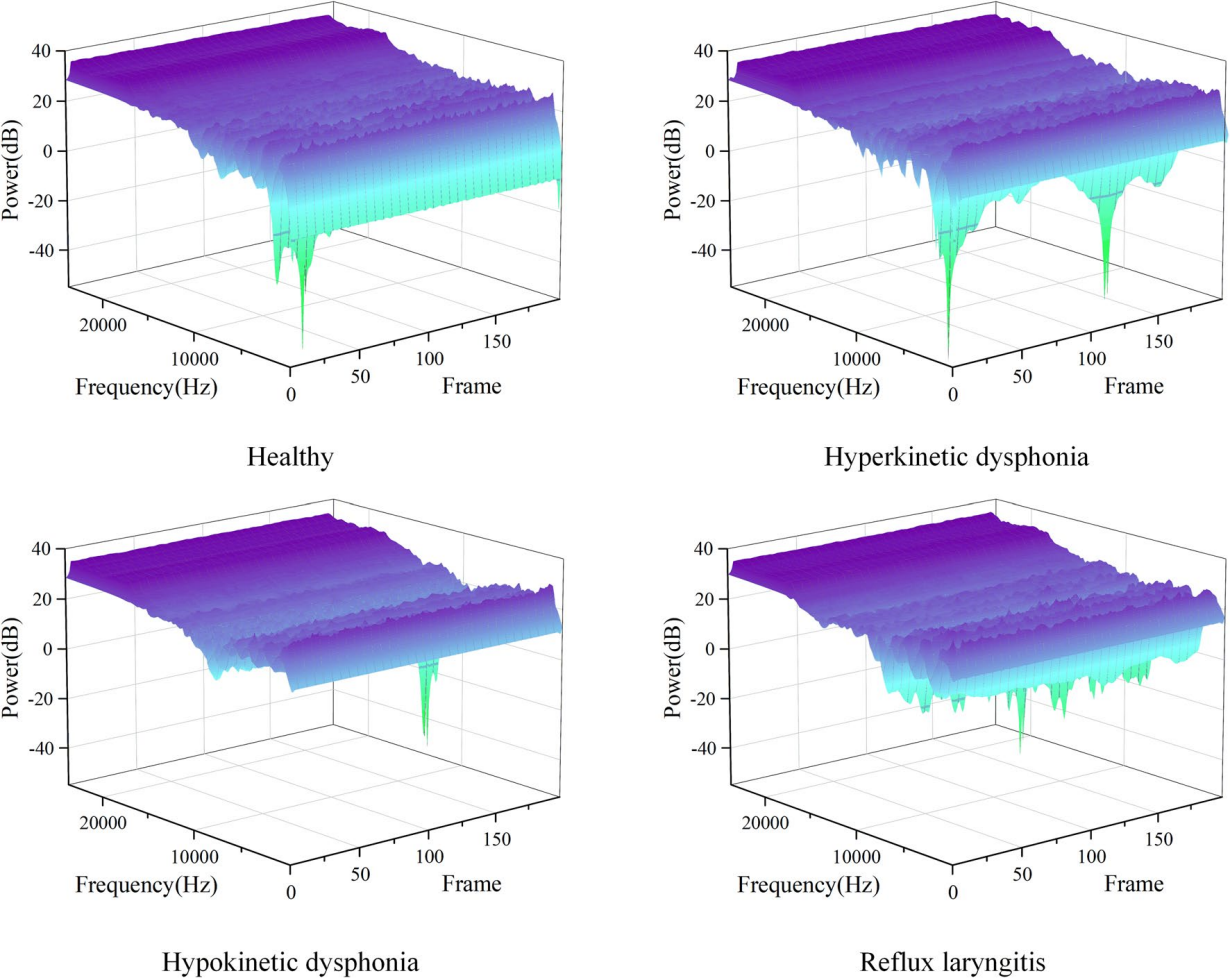
Mel spectra produced under different health states.

Since the signals are resampled to 48 kHz, according to the Nyquist sampling theorem, the highest frequency range of the filter is 24 kHz. We use a filter bank with 128 equal-area triangular filters, and the Fourier transform length is 2048 points. The 2048 points obtained by the Fourier transform obtain 128 energy values after passing through the Mel filter, which greatly reduces the amount of data. Each sample is divided into 199 frames. Each frame obtains 128 features through a Mel filter. The final dimensionality of the features that are input into the OpenL3 network is 199 × 128. The Mel energy spectra of the samples derived from different classes are shown in Fig. 3.

### High-level feature extraction based on the pretrained OpenL3 network

OpenL3 can automatically extract high-level feature embeddings from raw inputs. To verify the ability of OpenL3 to extract features from the raw data, t-distributed stochastic neighbor embedding (t-SNE)^33^ is used for feature visualization. t-SNE is a nonlinear dimensionality reduction technique that is well suited for the visualization of high-dimensional data.

**Figure 4.**
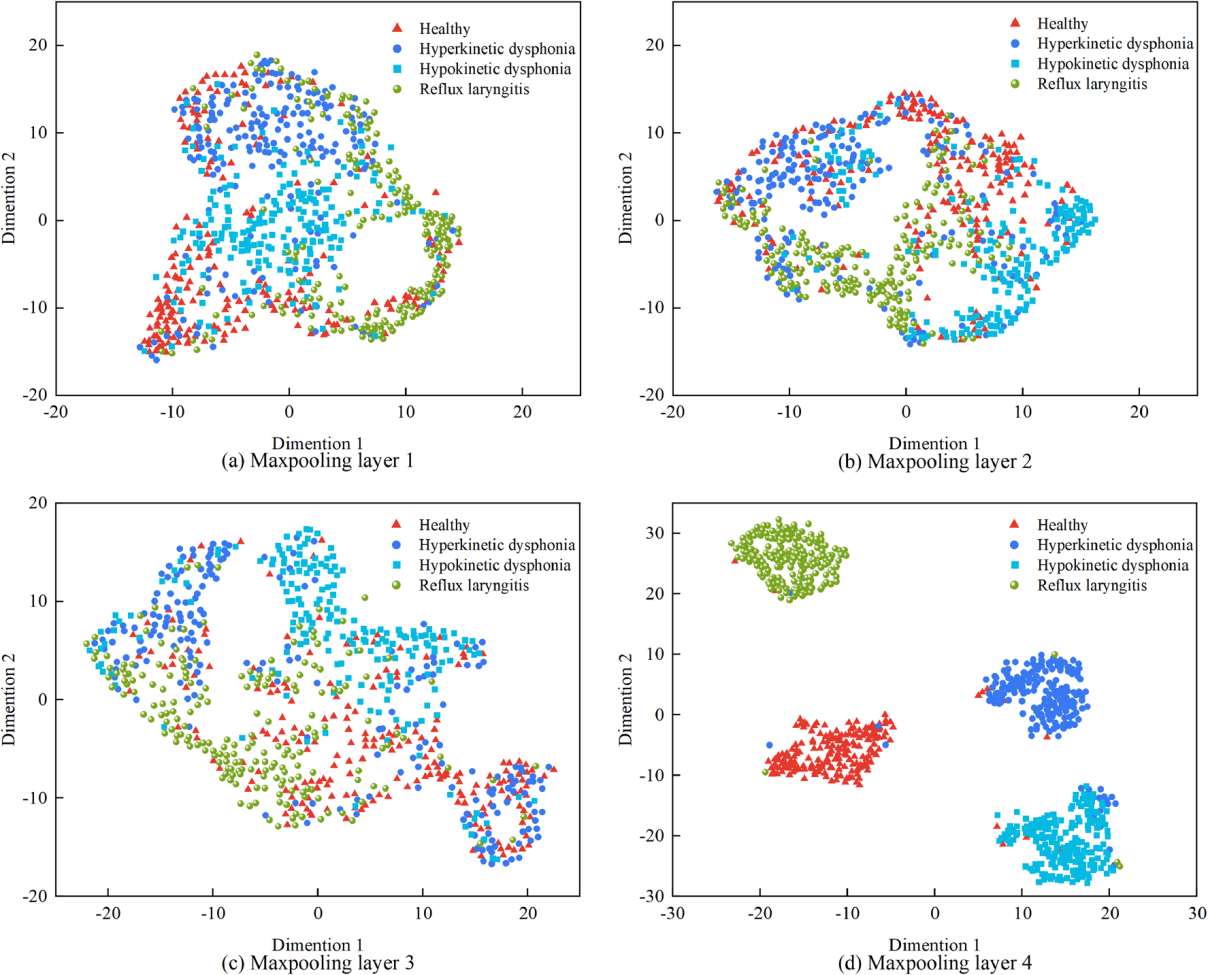
t-SNE visualization results obtained with the features of OpenL3.

To reproduce the process of OpenL3 extracting high-level features, the outputs of max pooling layers 1–4 are visualized and shown in Fig. 4. Figure 4a is max pooling layer 1, and there are no obvious boundaries between the samples of different classes. The outputs of max pooling layers 2 and 3 are shown in Fig. 4b and Fig. 4c, respectively. Although the distances between samples of different classes increase, the samples belonging to the same class do not exhibit a clustering status. Figure 4d is the output of max pooling layer 4. The samples of the same class are clustered into one cluster, and clear boundaries are observed between different samples. The feature visualization results prove that OpenL3 can learn high-level features with good discrimination from the raw input.

**Figure 5:**
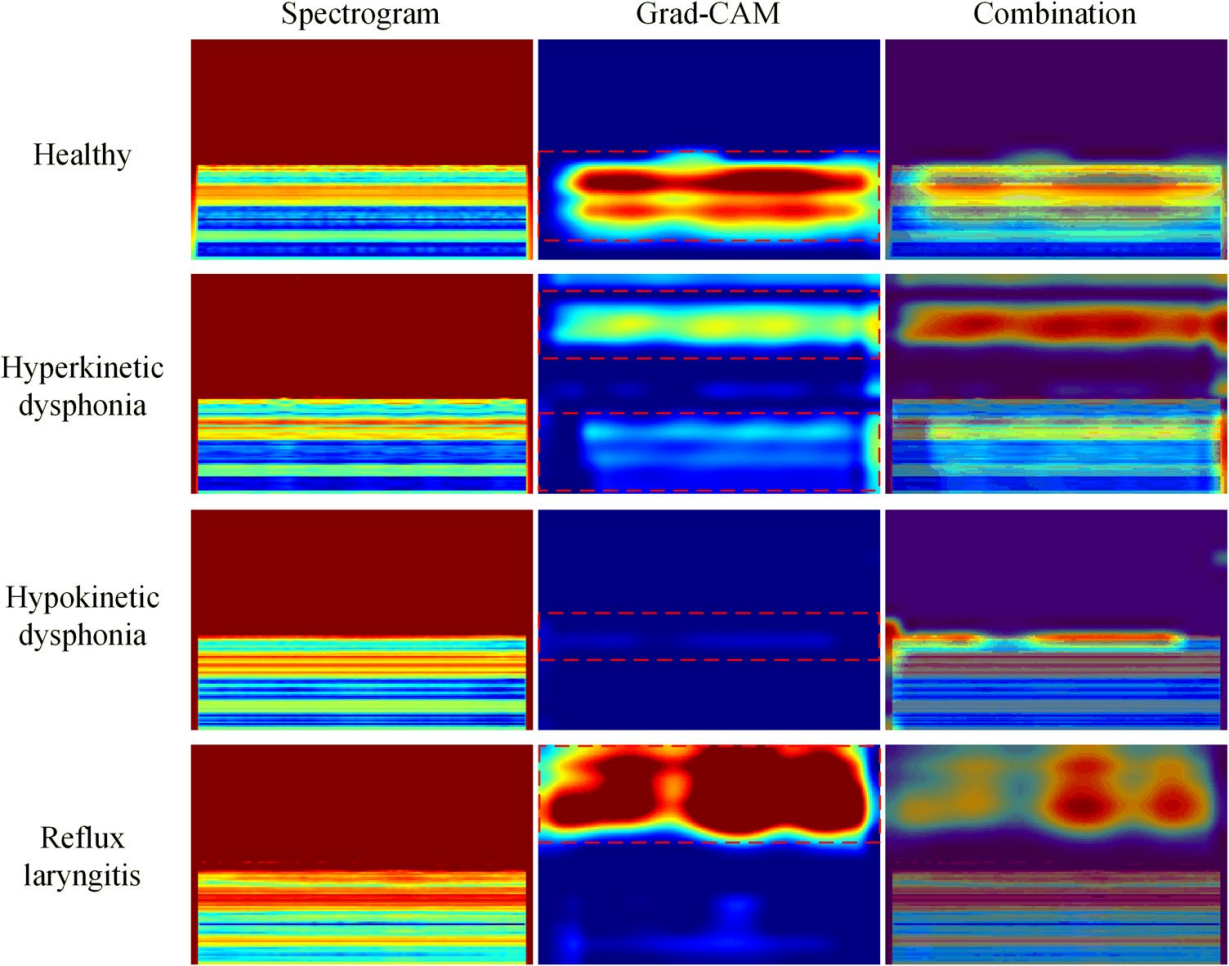
Grad-CAM results.

To reveal how the CNN performs in terms of feature learning, we visualize the region of interest of the model using gradient-weighted class activation mapping (Grad-CAM)^34^. Figure 5 presents the original spectrograms, the activation maps, and their combination for each class of samples. From the visualization results, it can be clearly observed that the model pays attention to specific features in different frequency bands depending on the different types of the input samples. For healthy samples, the model focuses on the low-frequency regions in the spectrogram. For hyperkinetic dysphonia samples, the model focuses on both the high-frequency and low-frequency parts. For the reflux laryngitis category, the model focuses on the high-frequency part of the spectrogram. However, for the hypokinetic dysphonia samples, the model does not bias its attention toward a certain region of the spectrogram and only has slightly higher activation values in the mid-frequency part than in the other regions.

512-dimensional feature vectors are extracted from each sample by OpenL3, but the high-dimensional vectors may contain some redundant features that are duplicated or do not contain important information. Redundancy in high-dimensional features not only increases the time required for network training but also hinders the network from finding data patterns. Therefore, we perform dimensionality reduction on the features extracted by OpenL3. In this paper, different feature dimensionality reduction methods are discussed under the OpenL3 framework.

To verify the effectiveness of feature dimensionality reduction, the raw features are compared with the dimensionally-reduced features. PCA^35^ is the most popular feature dimensionality reduction method, as it can preserve as much raw feature information as possible while mapping data from a high-dimensional space to a low-dimensional space. LLTSA^36^ is a manifold learning-based dimensionality reduction method that uses tangent spaces in the domain of data points to represent local geometry and then aligns these local tangent spaces into a low-dimensional space that is linearly mapped from the original high-dimensional space. mRMR^37^ is a feature selection algorithm that can find the set of features that are most correlated with the final output but least correlated with each other. ReliefF^38^ is a supervised feature selection method that assigns different weights to features based on their relevance to classes.

**Figure 6:**
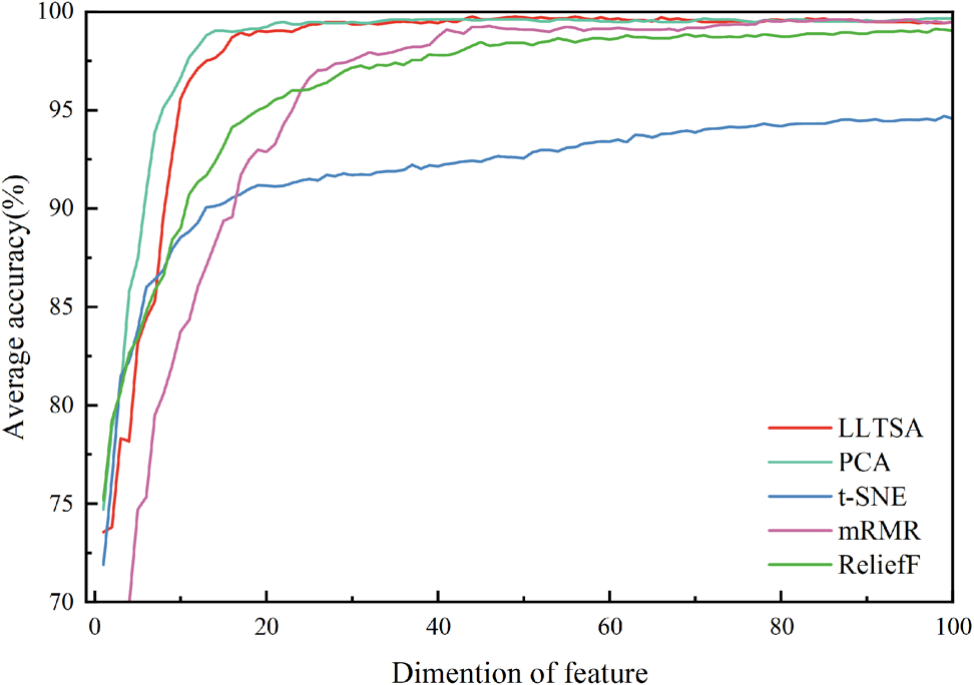
The effect of retaining different feature dimensions on accuracy.

We analyze the classification accuracy of each dimensionality reduction method when reducing the input to different dimensions. The experimental results are shown in Fig. 6. By observing where the accuracy increase converges as the dimensionality grows, we can determine how many feature dimensions each dimensionality reduction method should remove.

As shown in Fig. 6, the model using PCA and LLTSA dimensionality reduction has higher classification accuracy under the same dimensions. By observing where the classification accuracy growth converges, we finally determine the dimensions retained by each method, and the results are shown in Table 2. Following this, we conduct ten repeated experiments at these dimensions to validate the performance and stability of each method. The dimensions remaining after dimensionality reduction and the average performance across the ten experiments are shown in Table 2.

**Table 2:** The effect of different dimensionality reduction methods on the resulting accuracy.

Method	Dimensions	SEN (%)	SPE (%)	ACC (%)	F1 (%)
Raw features	512	98.6	95.4	97.9	98.6
PCA	**40**	99.5	98.7	**99.5**	99.5
ReliefF	60	99.2	97.5	98.8	99.2
mRMR	60	99.3	97.8	98.9	99.3
LLTSA	**40**	**99.6**	**98.9**	**99.5**	**99.6**
t-SNE	300	98.2	94.7	97.4	98.2

Significant values are in bold.

As seen from Table 2, utilizing LLTSA for dimensionality reduction yields superior model performance compared to that of the other methods. Although PCA and LLTSA achieve identical classification accuracies, LLTSA enhances the SEN by 0.1%, the SPE by 0.2%, and the F1 by 0.1%. The excellent performance of LLTSA may be attributed to the following two reasons. 1. As a dimensionality reduction method based on manifold learning, LLTSA maps high-dimensional data to a low-dimensional feature space while keeping the structure of the original data invariant. 2. The LLTSA algorithm takes the global and local structures of the dataset fully into account, which enables better clustering of irregular and inhomogeneous nonlinear data after performing dimensionality reduction. Consequently, LLTSA is chosen for feature dimensionality reduction in this paper.

**Figure 7:**
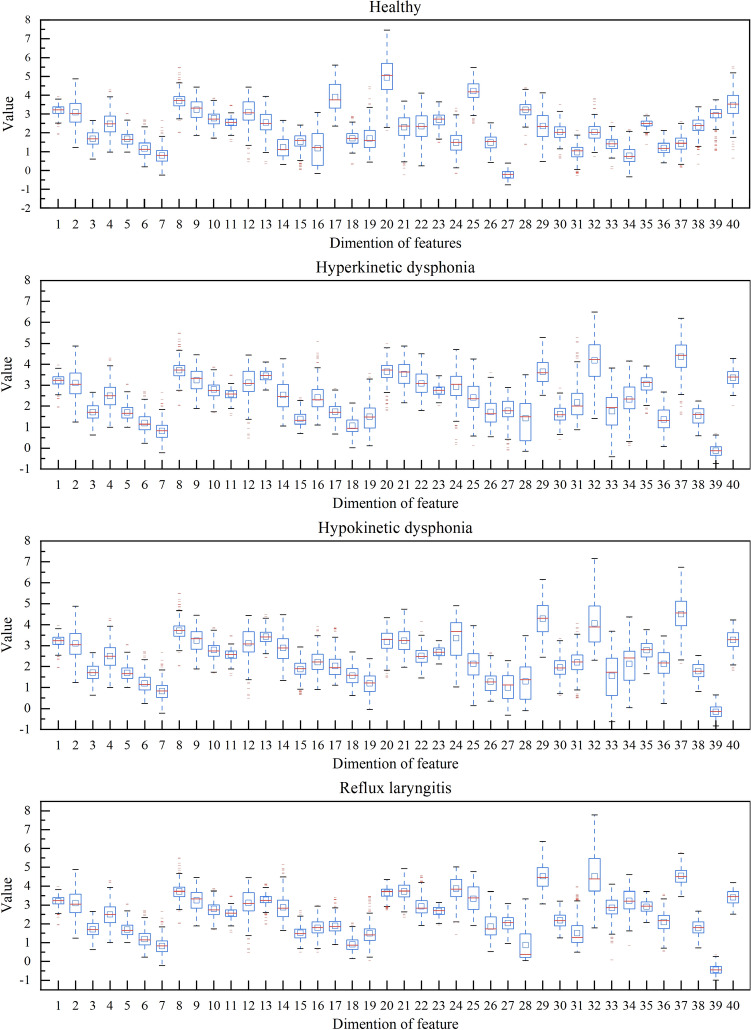
Boxplots of feature embeddings for different health states.

To analyze the feature distribution among the different classes, boxplots derived from different health states are shown in Fig. 7. The long red line in the middle of each box represents the median of the sample. The short red lines represent the outlier samples, and the top and bottom of each box represent the upper and lower quartiles, respectively. It can be observed from Fig. 7 that some outliers are contained in the feature embeddings, but their number is insignificant. Moreover, the distributions of the feature embeddings from different health states are dissimilar. These different feature embeddings provide the ability to accurately identify voice disorders.

### Performance comparison among the different pretrained models

Similar to OpenL3, VGGish^39^ is another pretrained model based on AudioSet. VGGish is capable of extracting 128-dimensional semantic feature vectors from audio waveforms. Several studies have indicated that the audio features obtained by the VGGish model are superior to artificially designed features provided by methods such as Mel, Constant-Q transform (CQT), and MFCC for various audio tasks. To verify the superiority of OpenL3, the end of the VGGish network is connected to an SVM classifier as a comparison model; this is similar to the structure of OpenL3-SVM. The performance comparison results are summarized in Table 3. As shown in Table 3, the network with OpenL3 as the embedding model significantly outperforms VGGish.

**Table 3:** Performance comparison among different pretrained models.

Model	SEN (%)	SPE (%)	ACC (%)	F1 (%)
OpenL3-SVM	**99.6**	**98.9**	**99.5**	**99.6**
VGGish-SVM	96.7	90.0	95.0	96.4

Significant values are in bold.

### The effects of different transfer strategies on performance

Not all transfers improve the performance of the network, and sometimes negative transfer may even occur. As the number of network layers increases, the computed features become increasingly relevant to the given dataset and task. Negative transfer can occur if a specific layer is transferred to a dataset that is significantly different from the source domain. Therefore, when using model-based transfer learning methods, different transfer strategies need to be adopted according to different transfer tasks. Three commonly used transfer learning strategies are presented as follows.

1. Freezing all: If the target dataset is small, retraining the deep network tends to cause overfitting. To circumvent this, all parameters of the pretrained model are frozen, and only the new fully connected layer and classification layer are trained for classification purposes. Alternatively, the output of the fully connected layer is directly extracted as a feature vector, upon which a new classifier is trained for classification.
2. Freezing and training: Generally, the shallow layers of the pretrained model can extract general features, and the deeper layers are suitable for specific tasks. Therefore, the parameters of the first n layers can be frozen, and the remaining layers can be fine-tuned. Typically, the fewer the number of network parameters and the smaller the dataset, the more layers must be frozen to prevent overfitting.
3. Retraining: If the target dataset is very large, overfitting does not easily occur during the training process involving the target dataset, and the parameters of the entire network can be retrained.

**Figure 8:**
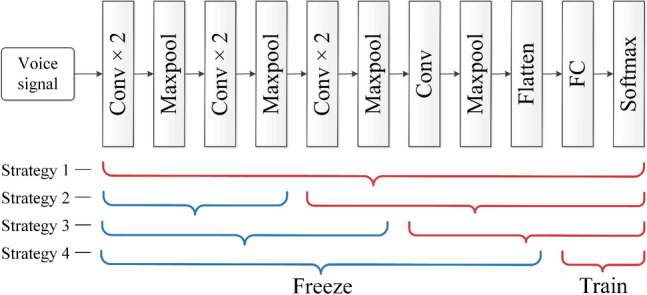
Transfer strategies for OpenL3.

According to the characteristics of the pretrained model, four transfer strategies are proposed for the voice disorder recognition task. The settings of these four strategies are shown in detail in Fig. 8.

To analyze the effects of different transfer strategies on performance, we conduct a series of experiments with different transfer strategies, and the results are summarized in Table 4. It can be seen from the experimental results that the fine-tuning of too many layers greatly reduces the performance of the model, and the accuracy achieved by retraining the entire network is only 94.4%, which is the lowest among all strategies. From strategies 1–4, as the number of frozen layers increases, the performance of the model first increases and then decreases, and the best performance is achieved when strategy 3 is adopted. There may be two reasons for this phenomenon. (1) Retraining a deep network on a small target dataset leads to severe overfitting; therefore, freezing more layers improves the model's performance. (2) AudioSet includes not only human voices but also environmental sounds and animal voices, which are not very similar to the target dataset. As the depth increases, deep neural networks tend to extract more specific features. Due to the difference between the source domain and the target domain, transferring the features of the last few layers may lead to negative transfer, so the performance of the model using strategy 4 degrades compared to that of the model using strategy 3.

**Table 4:** Performance comparison among different transfer strategies.

Transfer strategies		SEN (%)	SPE (%)	ACC (%)	F1(%)
Retraining	Strategy 1	96.3	88.8	94.4	96.3
Fine-tuning	Strategy 2	98.8	96.3	98.1	98.8
	**Strategy 3**	**99.6**	**98.9**	**99.5**	**99.6**
Freezing	Strategy 4	99.0	97.1	98.6	99.0

Significant values are in bold.

### Performance comparison

To further verify the effectiveness of OpenL3-SVM, OpenL3-SVM is compared with traditional machine learning and deep learning models, including a random forest (RF)^40^, an extreme learning machine (ELM)^41^, an SVM, a hierarchical extreme learning machine (H-ELM)^42^ and a deep sparse autoencoder (DSAE)^43^. Furthermore, we examine the necessity of utilizing the SVM to supplant the original Softmax classifier by evaluating the performance of OpenL3 with Softmax as the classifier. For the fairness of the experiment, all models are conducted under the same experimental conditions, each model is randomly run ten times through fivefold cross-validation, and the average values of all performance indicators are obtained as the final results. The main parameters of the above methods are set by the results of the grid optimization process, and the results are shown in Table 5.

**Table 5:** Main parameter settings of the tested methods.

Method	Main parameters	Value
OpenL3-SVM	Initial learning rate	0.001
	Learning rate drop factor	0.4
	Learning rate drop period	2
	Max number of epochs	10
	Minibatch size	32
	Penalty factor of the SVM	6.1
	Kernel parameter of the SVM	2.14
DSAE	Number of SAE1 nodes	15
	Number of SAE2 nodes	12
	L2 regularization weight of SAE1	0.5 × 10^–4^
	L2 regularization weight of SAE2	0.3 × 10^–4^
H-ELM	Number of ELM-SAE1 nodes	200
	Number of ELM-SAE2 nodes	300
	Sparse regularization weight	0.2 × 10^–29^
	Number of ELM hidden-layer nodes	500
RF	Number of decision trees	500
ELM	Number of hidden layer nodes	1000
SVM	Penalty factor for loss function	1
	Kernel parameter	4

The performance of the proposed OpenL3-SVM method is significantly improved compared to that of the original network using Softmax for classification, where the SPE is 10.65% higher than that of the original network. The superior performance of the SVM can be attributed to the following reasons. (1) In few-shot learning tasks, an SVM is less prone to overfitting than the Softmax function and can have a better generalization ability. (2) The CNN enhances the linear differentiability of linearly indistinguishable data during the process of convolution; therefore, the SVM can present the advantage of using only partial support vector samples for classification. Compared with the best comparison method (the RF), OpenL3-SVM improves the average SEN, SPE, ACC and F1 values by 1.2%, 3.7%, 1.8% and 1.2%, respectively.

**Figure 9:**
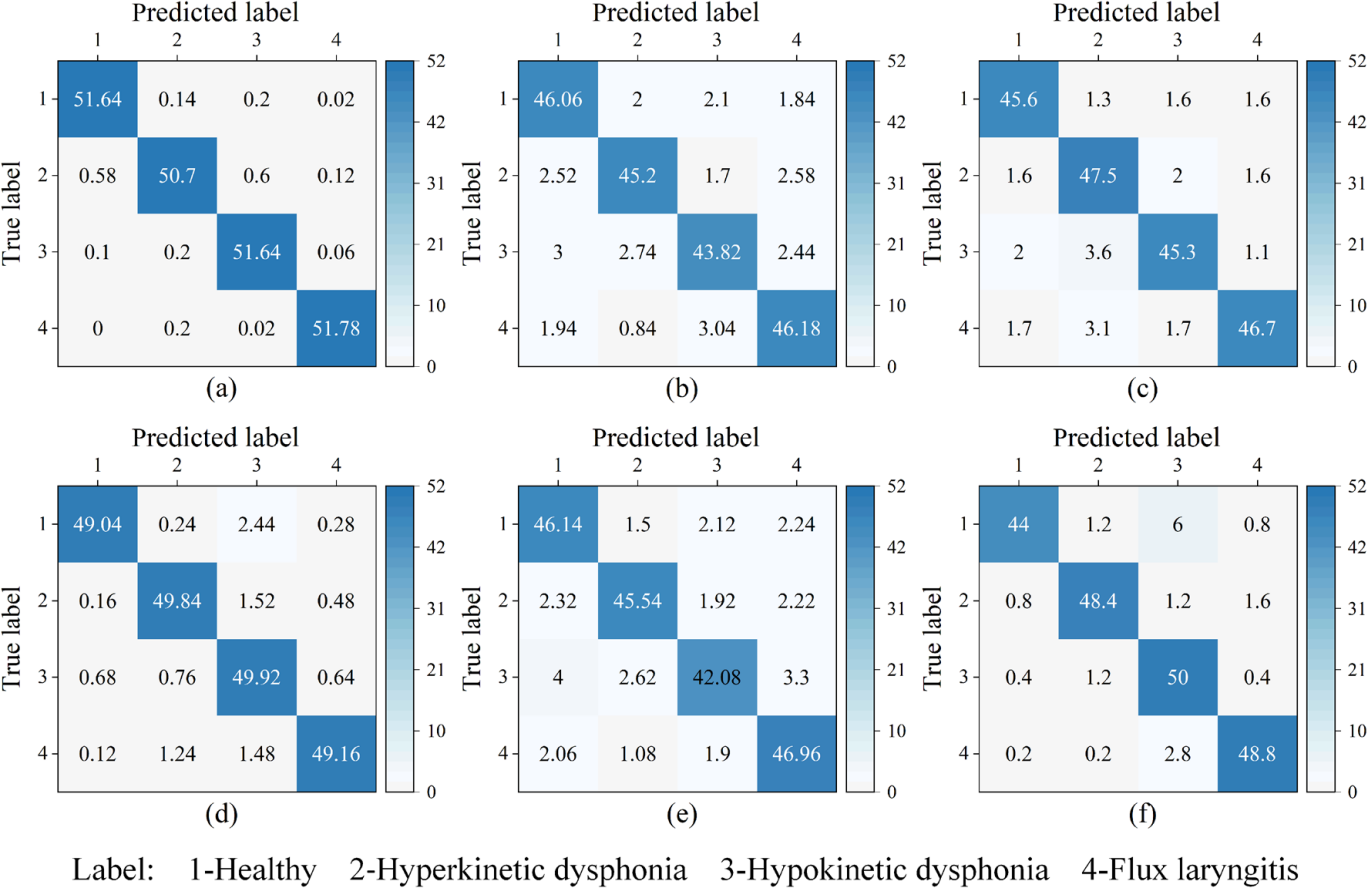
Average classification results of all methods. (**a**) OpenL3-SVM; (**b**) DSAE; (**c**) H-ELM; (**d**) RF; (**e**) ELM; (**f**) SVM.

We also present the classification results in the form of a confusion matrix in Fig. 9. To minimize randomness, we average the results of the fivefold cross-validation experiment for the presentation. The main diagonal represents the average number of samples that are correctly classified in each class, and the other positions represent the average numbers of misclassified samples. It can be seen from Fig. 9 that the average number of correct classifications for each class of produced by OpenL3-SVM for each class is significantly higher than that of the comparison method, which is also corroborated by the results in Table 6.

**Table 6:** Performance comparison.

Model	SEN (%)	SPE (%)	ACC (%)	F1(%)
OpenL3-SVM	**99.64** ± 0.21	**98.92** ± 0.63	**99.46** ± 0.31	**99.64** ± 0.21
OpenL3-Softmax	96.09 ± 0.94	88.27 ± 2.81	94.13 ± 1.41	96.09 ± 0.94
DSAE	95.71 ± 0.28	87.14 ± 0.85	93.57 ± 0.43	95.71 ± 0.28
H-ELM	96.33 ± 0.82	88.99 ± 2.47	94.50 ± 1.23	96.33 ± 0.82
ELM	95.63 ± 0.23	86.88 ± 0.68	93.44 ± 0.34	95.63 ± 0.23
SVM	97.31 ± **0.00**	91.92 ± **0.00**	95.96 ± **0.00**	97.31 ± **0.00**
RF	98.39 ± 0.10	95.17 ± 0.3	97.59 ± 0.15	98.39 ± 0.10

Significant values are in bold.

**Figure 10:**
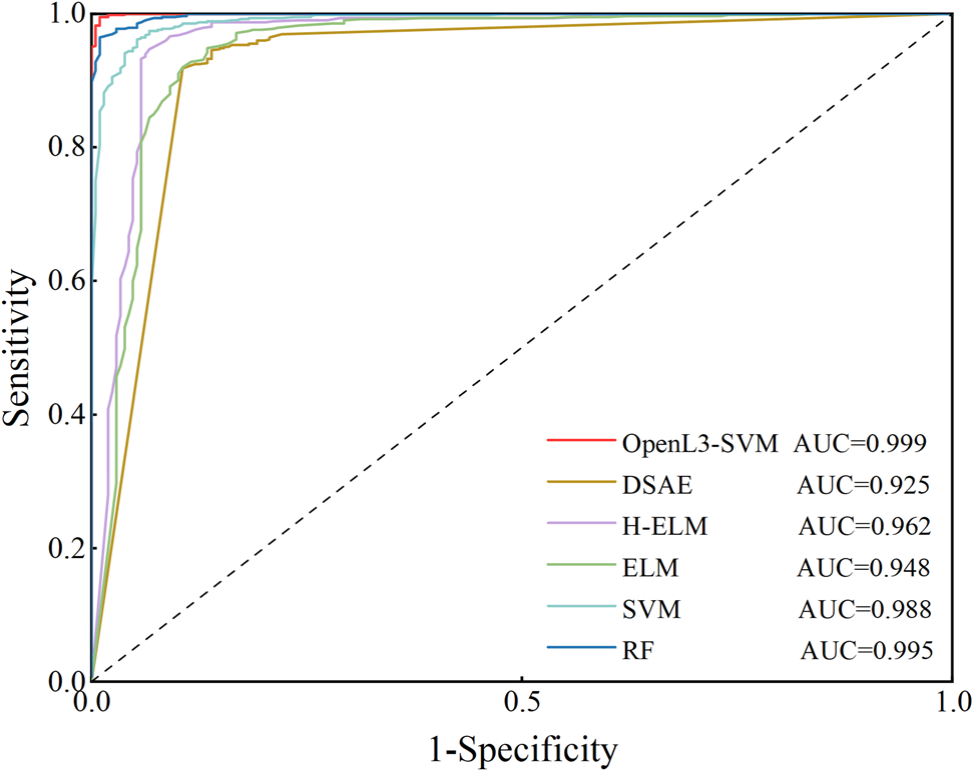
ROC curves of OpenL3-SVM and the compared methods.

The receiver operating characteristic (ROC) curve is also an effective way to evaluate model performance. The area under the ROC curve is called the AUC. The AUC is a well-recognized criterion for determining the overall performance of a model. A larger AUC value indicates better model performance. The ROC curves and AUC values of OpenL3-SVM and the comparative methods are shown in Fig. 10. As shown in Fig. 10, OpenL3-SVM, the RF and the SVM have the AUCs. This indicates that these three methods have the highest performance and robustness. Notably, the AUC of OpenL3-SVM is 0.999, which surpasses those of all other methods, highlighting its superior performance.

We also compare our method with recently proposed advanced methods, and the results are shown in Table 7. To ensure the fairness of the comparison, all methods are tested on the same dataset. Unified assessment metrics, including the ACC, SEN, and SPE, are employed; however, the accuracy of Kwok's method was not reported.

**Table 7:** Performance comparison with the published methods.

Author (year)	Method	Task Type	ACC (%)	SEN (%)	SPE (%)
Chen (2020)^17^	DNN	Binary classification	98.60	97.80	99.40
Mittal (2021)^18^	CGAN-IFCM	Binary classification	99.14	1	98.28
Kwok (2020)^19^	BiLSTM	Multiclass classification	–	90.10	89.40
Ours	OpenL3-SVM	Multiclass classification	99.46	99.64	98.92

The ACC of the proposed method reaches the highest value among those of all methods. The SEN and SPE values of Chen and Mittal's methods are higher than those of the proposed method, but both methods are based on a binary classification task. In comparison with Kwok's method, which is also based on a multiclass classification task, our approach achieves significantly higher SEN and SPE scores.

## Discussion

The direct application of CNNs is difficult due to the small amount of available data for the target task. By transferring knowledge from large-scale datasets based on transfer learning, the dependence of deep neural network training processes on sample size can be mitigated, resulting in improved performance for few-shot learning tasks. If a significant difference is present between the data distributions of the source and target domains, a fine-tuned transferring strategy may be required to optimize the model performance on the target task. In addition, a large number of redundant features may be extracted when pretrained models based on large-scale datasets are applied to few-shot learning tasks. Applying feature reduction techniques to the features extracted by the pretrained model can effectively speed up the training process without affecting the resulting classification performance.

Although OpenL3-SVM achieves good performance, only 208 recordings are used in this experiment. To enhance its performance, future research can expand the OpenL3-SVM training procedure to include a larger volume of voice recordings. This paper employs an under-sampling technique for dealing with class-imbalance data, which ignores some important sample information. In the future, we will investigate data augmentation techniques using adversarial neural networks to expand the class-imbalance data. With further performance improvements, it will become possible to use OpenL3-SVM as an auxiliary diagnostic tool for physicians.

## Conclusion

In this paper, a multiclass transfer learning framework for voice disorder classification is proposed. To explore the feature extraction process of the CNN, we perform a visual analysis using t-SNE and Grad-CAM. The results show that the utilized OpenL3 network can effectively extract sensitive high-level features from voice signals. Furthermore, the model pays attentions to specific features in different frequency bands depending on different types of samples. An SVM is employed instead of the original Softmax classifier, and the experimental results demonstrate that the SVM outperforms the Softmax classifier. To eliminate feature redundancy and accelerate the training process, LLTSA is used for feature dimensionality reduction. In addition, different transfer strategies are proposed and tested, and the results show that the fine-tuning strategy achieves the best performance. Through testing on the VOICED dataset, the proposed method achieves 99.46%, 99.64%, 98.92%, and 99.64% values for the ACC, SEN, SPE, and F1 metrics, respectively. Compared with the existing works and the compared machine learning methods, the proposed method exhibits better performance.

## Data Availability

The data that support the findings of this study are openly available in [PhysioNet] at https://physionet.org/content/voiced/1.0.0/, reference number^25^.
